# Upper limb arterial pattern: clinical correlation and embryological perspective

**DOI:** 10.1590/1677-5449.210008

**Published:** 2021-06-11

**Authors:** Laishram Sophia, Darshita Singh, Neha Xalxo, Anjoo Yadav, Sneh Agarwal, Urvashi Singh, Pooja Jain

**Affiliations:** 1 Lady Hardinge Medical College – LHMC, Department of Anatomy, Connaught Place, New Delhi, India.

**Keywords:** axillary artery, brachial artery, reconstructive surgery, angiography, coronary artery bypass, artéria axilar, artéria braquial, cirurgia reconstrutiva, angiografia, cirurgia de revascularização miocárdica com enxerto

## Abstract

**Background:**

Variations in the upper limb arterial pattern are commonplace and necessitate complete familiarity for successful surgical and interventional procedures. Variance in the vascular tree may involve any part of the axis artery of the upper limb, including the axillary artery and brachial artery or its branches, in the form of radial and ulnar arteries, which eventually supply the hand via anastomosing arches.

**Objectives:**

To study the peculiarities of the arterial pattern of the upper limb and to correlate them with embryological development.

**Methods:**

The entire arterial branching of forty-two upper limbs of formalin fixed adult human cadavers was examined during routine dissection for educational purposes, conducted over a 3-year period in the Department of Anatomy, Lady Hardinge Medical College, New Delhi.

**Results:**

The study found: 1) One case in which a common trunk arose from the third part of the axillary artery, which immediately splayed into four branches (2.4%); 2) High division of the brachial artery into ulnar and radial arteries, in 3 cases (7.1%); 3) Pentafurcation of the brachial artery into ulnar, interosseus, radial, and radial recurrent arteries and a muscular twig to the brachioradialis in 1/42 cases (2.4%); 4) Incomplete Superficial Palmar arch in 3/42 cases (7.1%); and 5) Presence of a median artery in 2/42 case(4.8%)

**Conclusions:**

This study observed and described the varied arterial patterns of the upper limb and identified the various anomalous patterns, supplementing the surgeon’s armamentarium in various surgical procedures, thereby helping to prevent complications or failures of reconstructive surgeries, bypass angiography, and many similar procedures.

## Resumo

**Contexto:**

As variações no padrão arterial dos membros superiores são comuns e, assim, necessitam de total familiaridade para que os procedimentos cirúrgicos e de intervenção sejam bem-sucedidos. A variância na árvore vascular pode envolver qualquer parte da artéria axial dos membros superiores, incluindo a artéria axilar, a artéria braquial ou os seus ramos, na forma das artérias radial e ulnar, as quais, em algum momento, suprem as mãos através dos arcos anastomosados.

**Objetivos:**

Avaliar as peculiaridades do padrão arterial dos membros superiores e correlacioná-las ao desenvolvimento embriológico.

**Métodos:**

Foram examinados os ramos arteriais completos de 42 membros superiores de cadáveres adultos conservados em formalina, os quais eram rotineiramente dissecados para fins educacionais durante 3 anos no Departamento de Anatomia Lady Hardinge Medical College, Nova Delhi.

**Resultados:**

O estudo apresentou cinco desfechos. 1. Foi constatado um caso em que um tronco comum surgiu da terceira parte da artéria axilar que imediatamente se disseminou em quatro ramos (2,4%). 2. Houve divisão maior da artéria braquial em artérias ulnar e radial em três casos (7,1%). 3. Em um caso, ocorreu pentafurcação da artéria braquial em ulnar, interóssea, radial, radial recorrente e de um galho muscular em braquiorradial (2,4%). 4. Foi constatado arco palmar superficial incompleto em três dos 42 casos (7,1%). 5. Foi observada a presença da artéria mediana em 2 dos 42 casos (4,8%).

**Conclusões:**

Este estudo compreende o padrão arterial do membro superior e identifica os diversos padrões anômalos para agregar ao arsenal terapêutico de cirurgiões para diversos procedimentos cirúrgicos, com o objetivo de combater quaisquer complicações ou falhas de cirurgias reconstrutivas, de angiografias de cirurgias de revascularização e muitas outras.

## INTRODUCTION

The web of arterial patterns in the body has always been an enigma. Inconsistencies in the arterial pattern of the upper limb in both origin and distribution are the rule rather than an exception. The axillary artery (AA) is a direct continuation of the subclavian artery, extending from the outer border of the first rib to the lower border of the teres major and thereafter continuing as the brachial artery (BA) of the upper limb.^1^ For the purposes of description, the AA is divided into three parts by the pectoralis minor, which passes over it, and a total of six branches arise from it: namely the superior thoracic artery from the first, the thoraco-acromial (TAA) and lateral thoracic arteries (LTA) from the second, and the subscapular (SSA), anterior circumflex humeral (ACHA), and posterior circumflex humeral arteries (PCHA) from the third part. Although the classic six branches from the AA are invariably described in every textbook, there are countless publications in the literature about the diverse nature of its multiple branches. The typical version of six branches originating distinctly from the axillary artery is actually seen in only 27% of cases.^2^ The extensive collateral circulation between the subclavian artery and the third part of the AA makes variations of the axillary artery clinically important. This article depicts the variation in the arterial pattern of the upper limb and describes previously unreported branching patterns of the AA and brachial artery (BA), each of which was found in one of the 24 embalmed cadavers dissected at our institution, discussing their embryological and clinical significance.

## MATERIALS & METHODS

### General information

This observational study was conducted with donated human cadavers meant for research work and teaching for first-year Bachelor of Medicine and Bachelor of Surgery undergraduates at the medical institution. Eight to nine embalmed cadavers are designated for dissection purpose in the research laboratory for each new academic year and, since the study was conducted over a 3-year time frame, 24 embalmed cadavers and a total of 42 upper limbs were included. Cadavers that were not appropriate were excluded from the study. The Local Government approved the ethical aspects and policy for conducting research studies and for teaching purpose on donated human bodies at the Department of Anatomy in the Government Medical institution. Informed written consent was also obtained at the time of body donation from the donors themselves or their relatives.

### Methods

Cunningham’s Manual of Practical Anatomy^3^ was followed throughout the whole dissection procedure, taking care to preserve all the arteries, while sacrificing venae comitantes. Photographs were taken with a Canon EOS 7D Mark II DSLR and labeled using GoodNotes software.

## RESULTS

In the present study on 42 upper limbs (Table 1), the arteries and their branching pattern were keenly observed and all variations were noted.

**Table 1:** Comprehensive overview of the results obtained during the study.

No.	Variant Artery	No. of cases/total no. of cases	percentage
1	Axillary artery	1/42	2.4
2	High brachial artery division	3/42	7.1
3	Pentafurcation of brachial artery	1/42	2.4
4	Ulnar artery (Superficial Palmar Arch)	3/42	7.1
5	Median artery	2/42	4.8

**Figure 1:**
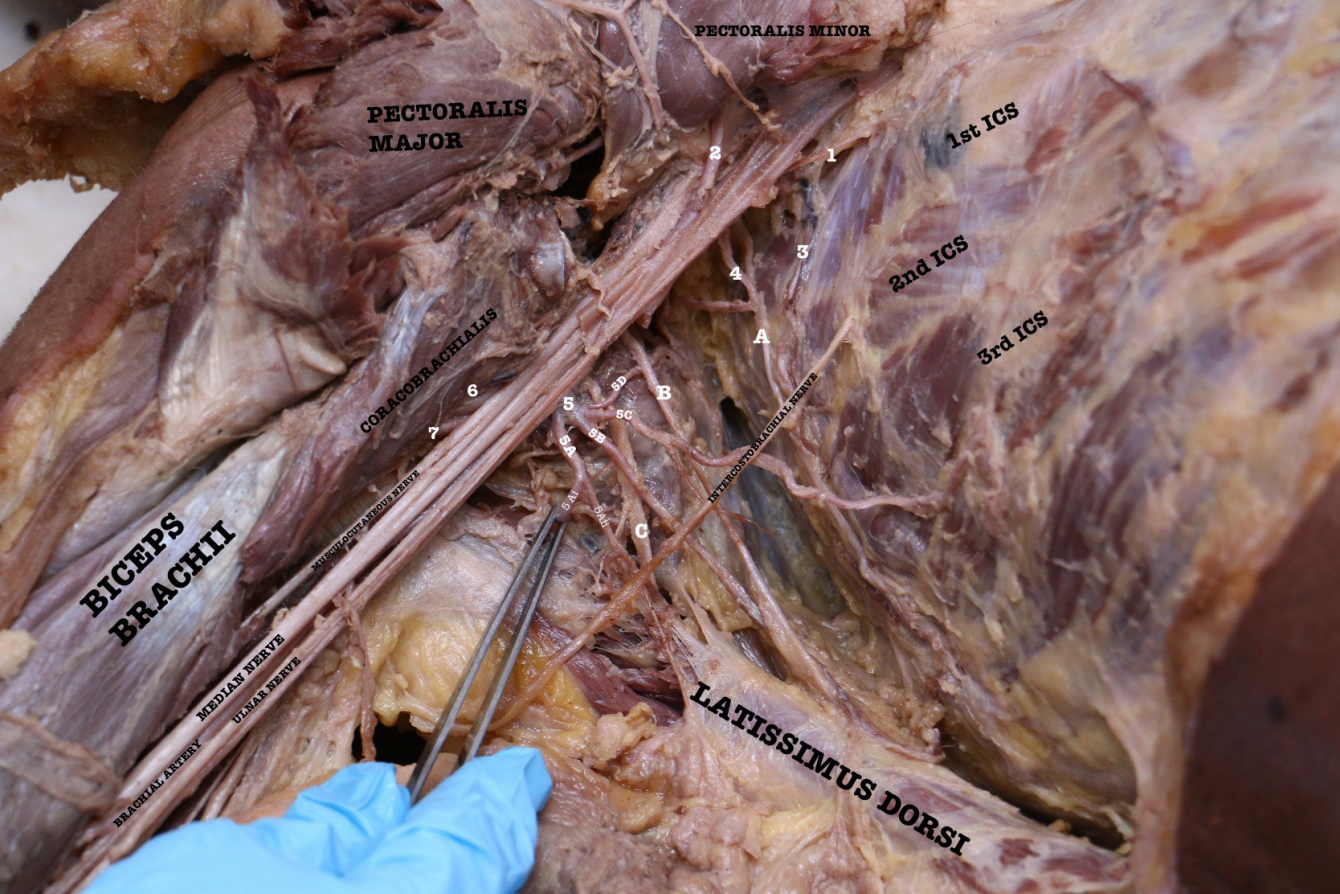
Dissected right axilla. (1) Superior Thoracic Artery; (2) Thoracoacromial Trunk; (3) Lateral Thoracic vessels; (4) Alar Thoracic Artery; (5) Common trunk; (5A) Subscapular Artery; (5Ai) Circumflex Scapular Artery; (5Aii) Thoracodorsal Artery); (5B) Thoracodorsal artery; (5C) additional Lateral Thoracic Artery; (5D) muscular branch to Subscapularis; (6) Anterior Circumflex Humeral Artery; (7) Posterior Circumflex Humeral Artery; (A) Long Thoracic Nerve; (B) Thoracodorsal Nerve; (C) Lower Subscapular Nerve (ICS – Intercostal Space).

In one of the 42 cases, an extremely rare type of variation was seen involving the axillary artery (second and third part, [Figure 1]). The first part was absolutely as per the description given in the standard textbooks, i.e. with a single artery, the superior thoracic artery. The second part had three branches instead of the usual two, specifically, the thoracoacromial artery, the lateral thoracic artery, and an alar thoracic artery. From the third part, once again three branches were observed, specifically, the anterior circumflex humeral artery (ACHA), the posterior circumflex humeral artery (PCHA), and a third branch which in fact was a large common trunk (which later tetrafurcated) at the anatomical position of the subscapular artery (SSA [Figure 1]). This common trunk gave rise to the following arteries:

1. Subscapular artery (SSA [Figure 1, labeled 5A]), which divided into the Circumflex Scapular artery (CSA [Figure 1, labeled 5Ai]), winding around the lateral border of scapula in the upper triangular space, and a Thoracodorsal Artery (TDA [Figure 1, labeled 5Aii]);
2. Thoracodorsal artery (TDA), supplying the latissimus dorsi muscle along with the thoracodorsal nerve (Figure 1, labeled 5B);
3. An additional lateral thoracic artery (LTA) supplying the lateral thoracic wall especially the third and fourth intercostal areas including the respective serratus anterior muscle parts and mammary gland (Figure 1, labeled 5C);
4. A muscular branch to the subscapularis (Figure 1, labeled 5D).

**Figure 2:**
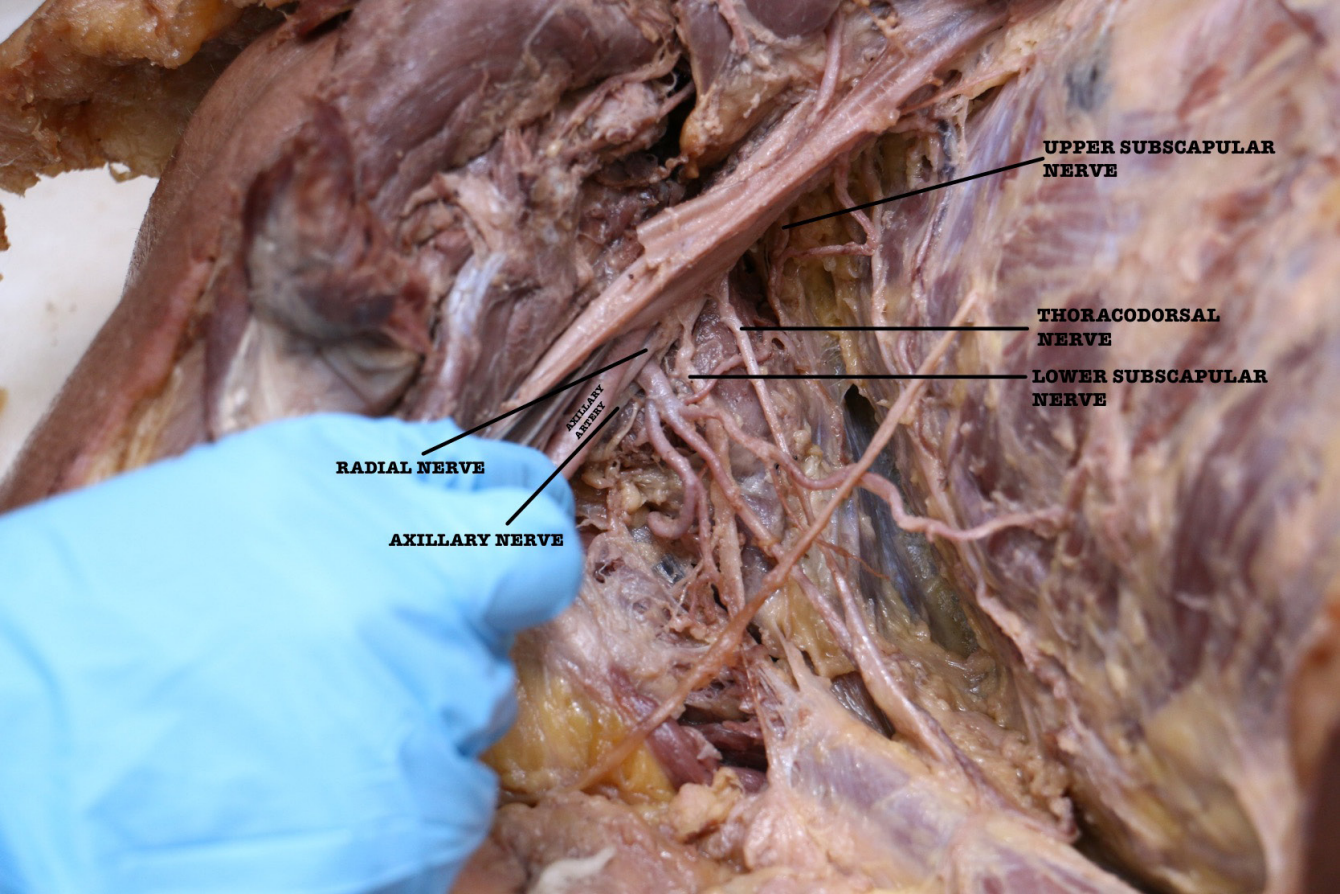
Branches of the posterior cord surrounding the common trunk of the third part of the axillary artery. Axillary nerve passes posteriorly and radial nerve anteriorly.

Occasionally, the branches of the brachial plexus surround the common vessels instead of the regular axillary artery.^1^ In this case, branches of the posterior cord of the brachial plexus surrounded the common trunk, with the axillary nerve passing posteriorly and the radial nerve anteriorly (Figure 2).

**Figure 3:**
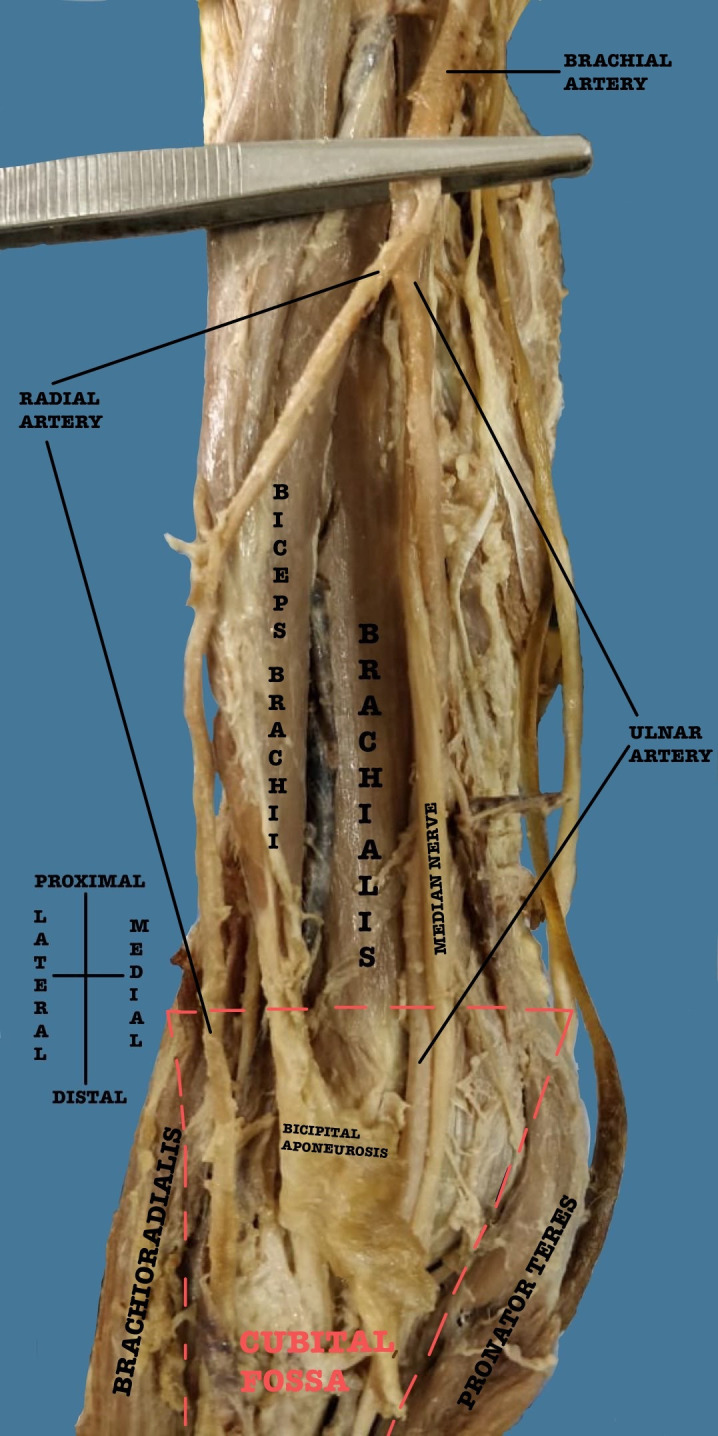
High division of the brachial artery into radial and ulnar artery.

The brachial artery also showed variations in four out of forty-two cases. In three cases, a high division of the brachial artery into radial and ulnar arteries was seen. These high divisions were measured with respect to the intercondylar line of the humerus and were seen at 13 cm, 14.5 cm, and 15 cm above the intercondylar line respectively (Figure 3).

**Figure 4:**
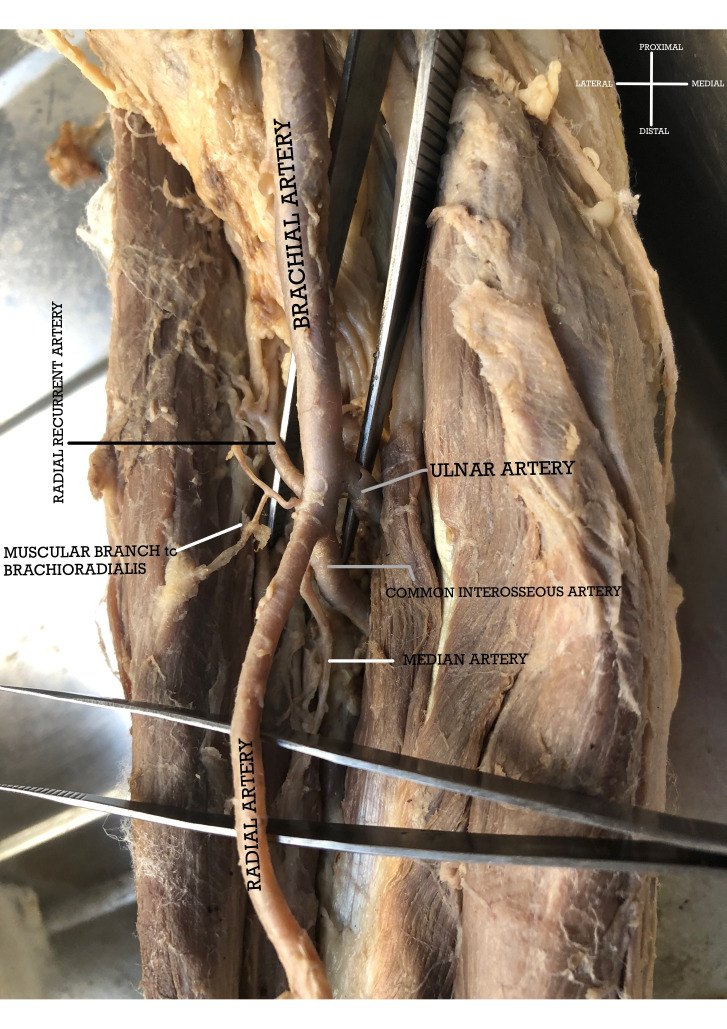
Pentafurcation of the brachial artery (from medial to lateral) into Ulnar Artery, Common Interosseous Artery, Radial Artery, Muscular branch to Brachioradialis, and Radial Recurrent Artery. [N.B. a persistent Median Artery arises from the Common Interosseous Artery].

In the fourth case a previously undescribed pentafurcation of the brachial artery was seen. The divisions were into radial artery, ulnar artery, common interosseous artery, radial recurrent artery, and a muscular branch to the brachioradialis muscle (Figure 4).

The common interosseous artery (CIA) gave rise to the usual anterior and posterior interosseus artery and a persistent median artery, which passed deep to the pronator teres and accompanied the median nerve.

**Figure 5:**
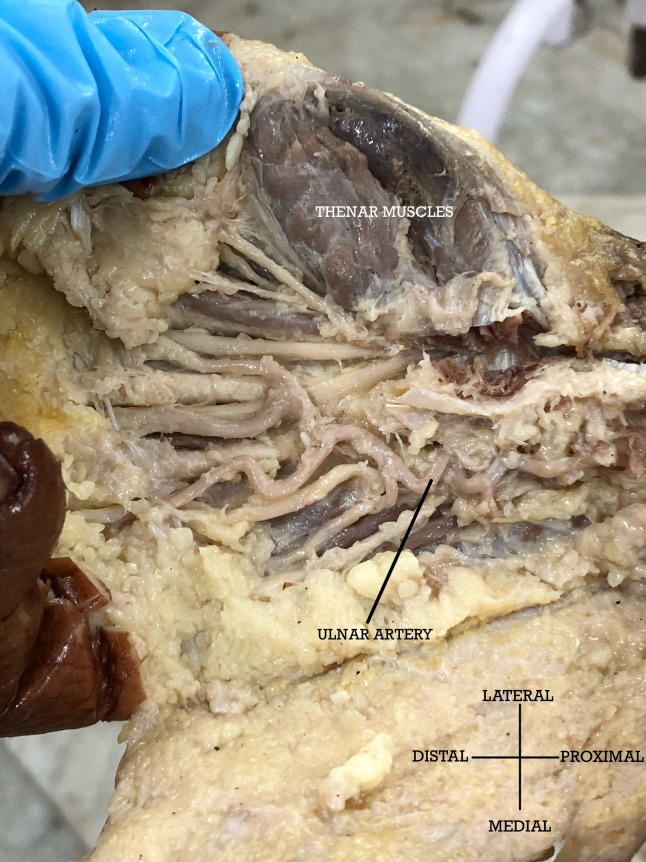
Tortuous Ulnar Artery in the hand with sole participation in the Superficial Palmar Arch, which has no contribution from the Radial Artery.

The superficial palmar arch (SPA), which is completed by a superficial palmar branch of the radial artery and infrequently by an arteria princeps pollicis or arteria radialis indices, was not evident in three out of 42 limbs. In these cases, there was no contribution from a superficial branch or any other branch from the radial artery and therefore an incomplete SPA was observed that was formed solely by the ulnar artery in the palm (Figure 5). A median artery was seen in two cases.

## DISCUSSION

Deviations of the upper limb arterial pattern from its anatomical norms have been frequently reported, which makes it even more essential to familiarize one with the possible patterns for successful surgical interventions.

Several authors have reported an exceptional number of branches from the axillary artery varying from seven to eight branches to as many as five to eleven branches.^1^ Authors have classified the AA into twenty-three types on this basis.^4^

A very close similarity to our finding of variant AA is presented in a case report on the tetrafurcation from the third part of the AA, where a common trunk gave origin to TDA, CSA, PCHA, and LTA, whereas, in our study the PCHA had a normal origin from the third part of the axillary artery and the common trunk gave rise to an auxiliary LTA and the TDA, in addition to the SSA branch itself, which divided into CSA and TDA.^5^

The SSA is known to supply the subscapularis muscle by giving off one collateral branch in 31.1% and two branches in 29.3%.^6^ A similar finding was also seen in our study.

In another study conducted on 40 cadavers, the LTA emerged from the SSA^7^ but there was no additional LTA reported, unlike in our study. However, in a study of 420 cadavers, the percentage of multiple LTAs was 3.09%, which is similar to our study, in which the single case equated to 2.4% and was classified as type V on the basis of its origin.^8^

The TDA defines the posterior boundary of axillary lymph node dissection and any derailment of its normal course can compromise oncology results in surgeries involving lymph node dissections. The presence of multiple TDA seen in our study renders isolation of the vascular pedicle for free flap transfer a perilous task and could result in postoperative complications or failure of the flap itself.

There are considerable variations of the brachial artery (BA) documented in the literature. In the majority, the brachial artery, which is a continuation of the axillary artery, begins at the inferior border of the teres major and ends by bifurcating about a centimeter distal to the elbow joint into radial and ulnar arteries. The two main classifications of arterial arm variations include one in which a brachial artery is positioned anterior to the median nerve, described as the superficial brachial artery, while the other main anomaly involves doubling of the brachial artery into superficial and deep brachial arteries. The former is called the superficial brachial artery superior when it occurs at the median nerve roots in the axilla. Another version of the superficial brachial artery is positioned posterior to the median nerve, but ultimately travels anterior to the median nerve more distally in the arm.^9^

In cases where the brachial artery is doubled or presents with high bifurcation of the vessel, the superficial and deep brachial artery reunite distally before eventually either dividing into radial and ulnar artery or continuing as only a radial or an ulnar artery, with or without collateral branches.^9^ The superficial brachial artery can also persist as the median artery or interosseous artery. Doubling of the brachial artery can be simply early bifurcation into radial and ulnar arteries near median nerve roots.^10^ The majority of superficial brachial arteries often continue as the radial artery. Its continuation as a superficial ulnar artery (SUA)^11^^,^ 12 and as a superficial brachioradial artery (high origin of the radial artery) have also been reported, stemming from the axillary artery.

Unlike in our case, trifurcation of the brachial artery has been documented in the literature, dividing into radial, ulnar, and superior collateral arteries;^13^ radial, ulnar, and muscular branches,^14^ and radial, ulnar, and common interosseous arteries.^15^ No cases have been documented in which the brachial artery divided into five branches.

The brachial artery is reported to become compressed by the bicipital aponeurosis in athletes with hypertrophied muscles in the forearm, to the extent that it causes discomfort and even obliterates the radial pulse.^16^ The multiple division of the BA in the cubital fossa seen in our study would cause immense damage to the arterial supply of the antebrachium if such a condition existed.

In our study, one cadaver had a median artery (MA) that originated from the common interosseous artery and travelled deep to the pronator teres, supplying the brachioradialis and accompanying the median nerve. The median artery did not pass under the carpal tunnel. The RA and the MA ended in the musculature of the forearm, making no significant contribution to the hand. As a result, an incomplete superficial palmar arch with no participation from the radial artery was observed in the hand. It was further noted that the ulnar artery that was solely involved in the superficial palmar arch was highly tortuous. The tortuosity of the ulnar artery is explained due to involvement of the ulnar nerve as an occupational hazard in those who engage in constant hammering/vibrating actions and repetitive traumatic activities; a condition known as hypothenar hammer syndrome.^17^ In turn, the tortuous ulnar artery can lead to Guyon’s canal syndrome.^18^

### Clinical significance

Unwanted vascular injuries can be circumvented during surgical and other interventional procedures if any vascular anomaly is delineated in advance by Doppler study or MRI with angiography. The thoracodorsal and circumflex scapular vessels are used as recipient sites for autologous microsurgical reconstructions with free flaps such as deep inferior epigastric artery perforator (DIEP) or profunda femoris artery perforator (PAP) flap procedures.^19^^-^ 21 Subscapular and thoracodorsal vessels denote the posterior extent of axillary lymph node removal procedures following positive sentinel node dissection in invasive breast cancers. The axillary artery branches are also used to create axillary-coronary bypass shunts in high-risk patients. Orthopedic surgeons attempting delayed reduction of a dislocation must have a thorough knowledge of the branching pattern anomalies, especially when the artery is adherent to the articular capsule.^1^ The AA tree is necessary during antegrade cerebral perfusion in aortic surgery,^22^ in usage of medial arm skin flap,^23^ for treatment of axillary artery thrombosis,^24^ for reconstruction of the AA after trauma, and in treatment of axillary artery hematoma and brachial plexus palsy.

Due to long term patency and survival benefits, the radial artery is a versatile conduit for CAB (coronary artery bypass) and open radial artery harvesting reduces the risk of endothelial damage.^25^^,^ 26 Although bilateral internal thoracic artery (BITA) grafting is the method of choice, RA is still considered in cases in which use of the second internal thoracic artery (ITA) is not feasible due to diabetes, obesity, COPD, or sternal conditions.^27^ The variant in which the recurrent radial artery does not arise from the RA, as presented in our case, could perhaps ease the trans-radial approach for endovascular neurointervention, which otherwise could impede success in cases in which a radial loop or recurrent radial artery exist.^28^

Considering the plethora of clinical significance scenarios, it is imperative for clinicians to have a thorough knowledge of the normal and aberrant vessels in the branching pattern of the upper limb.

### Embryological basis

The principal arteries anastomose and periarticular networks of capillaries emerge according to a temporal sequence; some paths that are initially functionally dominant subsequently regress. The anomalous patterns occur as differences in the mode and proximodistal level of branching, aberrant vessels anastomosing with principal vessels, and/or vessels forming unexpected neural, myological, or osteoligamentous relationships.^1^ The type of variation depends on the differentiation, enlargement, and persistence of the initial plexus of the capillaries.^29^ Such types of variation may perhaps be due to defects in the proximal part of the lateral branch of the seventh cervical intersegmental artery.^30^ The pattern of the blood vessels is to some extent predetermined by genetics.^31^ Genetics also plays a key role in early embryogenesis, determining the identity and positioning of blood vessels, whereas in the later part of embryogenesis, blood flow influences remodeling of the blood vessels.^32^

In conclusion, understanding of the normal and variant patterns of the vascular system is imperative for a favorable postsurgical outcome when the surgeon is taking an on-table decision in vessel selection for reconstructive surgery. Rarer variations are encountered more frequently than anticipated. Therefore, having a thorough knowledge about the kinds of variants that exist might help to raise the level of suspicion and thus extend the reach of the safety net for preventing surgical catastrophes. The present study is intended to raise awareness in the minds of surgeons and radiologists alike about the variations of the arterial arrangement of the upper limb that could determine the outcomes of surgical and interventional procedures.
